# Good to Excellent Functional Outcome and High Return to Sports Rate after Operative Treatment of Unstable Lateral Clavicle Fractures: Comparison of Two Coracoclavicular Button Fixation Techniques

**DOI:** 10.3390/jcm10204685

**Published:** 2021-10-13

**Authors:** Markus Wurm, Michael Zyskowski, Sebastian Pesch, Peter Biberthaler, Chlodwig Kirchhoff, Marc Beirer

**Affiliations:** Department of Trauma Surgery, Klinikum Rechts der Isar, Technical University Munich, 81675 Munich, Germany; markus.wurm@mri.tum.de (M.W.); michael.zyswkoski@mri.tum.de (M.Z.); sebastian.pesch@mri.tum.de (S.P.); peter.biberthaler@mri.tum.de (P.B.); marc.beirer@tum.de (M.B.)

**Keywords:** lateral, clavicle, fracture, arthroscopy, ORIF, return to sport

## Abstract

Purpose: Operative therapy for unstable lateral clavicle fractures is necessary to reduce the risk of bony non-union. Irritation and restriction during sportive activities due to the implanted materials are a common reason for impaired function and implant removal. The aim of this study was to gain information on functional outcome and time until return to sport (RTS) after surgical treatment of unstable lateral clavicle fractures, comparing two coracoclavicular button techniques. Methods: A retrospective chart review of patients who were consecutively treated for unstable lateral clavicle fractures at our level one trauma center from 2014 to 2018 was conducted. Two different surgical techniques were evaluated and compared. Group 1 was treated using a locking compression plate and knotted DogBone™ Button, while group 2 received an LCP and knotless DogBone™ Button. Functional outcome (ASES (American Shoulder and Elbow Score), Constant-Score, DASH (Disability of Arm, Shoulder and Hand), MSQ (Munich Shoulder Questionnaire) and SPADI (Shoulder Pain and Disability Index) and time until RTS were investigated and compared between both groups, 1 year postoperatively. Results: A total of 56 patients (*n* = 35 group 1, *n* = 21 group 2) with a mean age of 45.1 ± 14.6 years met the inclusion criteria. Functional outcome reached good to excellent results (ASES 94.7 ± 9.8, Constant Score 85.1 ± 8.1, DASH 5.5 ± 8.4, MSQ 90.9 ± 7.2, SPADI 96.1 ± 5.7). Implant removal rates were higher in group 1 (48.3% vs. 35.3%) yet without statistical significance (*p* = 0.122). All patients returned to sports postoperatively with a mean time period until return to sport of 4.6 (3–9) months. Conclusion: Locking compression plating and coracoclavicular fixation using a knotless Dogbone™ technique provides good to excellent functional outcomes, a high and fast rate of return to sport and lower irritation rates compared to the knotted DogBone™ technique.

## 1. Introduction

Unstable lateral clavicle fractures are prone to mal- or non-union when treated conservatively [1,2]. In 1968, Charles Neer reported on inferior functional outcome in conservatively treated patients with unstable lateral clavicle fractures [3]. These fractures are nowadays commonly classified using the Neer classification, which distinguishes stable (type I) from unstable fractures (type IIa, IIb) needing operative treatment. In this context, coracoclavicular ligament injury leads to vertical instability of the lateral clavicle potentially, resulting in mal- or non-union when treated non-operatively. Functional outcomes of conservatively treated patients showed controversial results reaching from bad results in young and active patients to good, medium-term results in mid-aged patients [4,5]. However, operative management revealed better functional outcomes and less mal- or non-union [5,6]. Not only unstable but also displaced fractures are in risk of mal- or non-union. Therefore, the degree of displacement (>100% shaft displacement) or vertical and/or horizonal instability is considered as an indication for operative treatment [7,8]. In this context, hook plating, as well as the arthroscopic assisted button technique are two widely accepted procedures which provide good to excellent results for this entity in the common literature [9,10]. Biomechanical testing of different surgical techniques revealed comparable results with respect to load-to-failure [11], although the LCP (locking compression plate) technique with an additional button procedure was proven to be more stable [11,12]. However, hook plating presented with higher rates of implant related complications, therefore the arthroscopic technique has become the routine treatment technique for unstable lateral clavicle fractures in our trauma department [2]. Nevertheless, implant related irritation also occurred in patients who were treated by the arthroscopy assisted technique. Therefore, a knotless technique has been introduced to avoid irritation, presumably caused by fibers of the knotted technique. To the best of our knowledge, there is no study in current literature so far addressing this problem. Implant related irritation during daily as well as sporting activities leads to restrictions which are often followed by patients’ wish for implant removal [13,14]. There are only few data on return to sports (RTS) after ORIF (open reduction-internal fixation) of lateral clavicle fractures in the literature. However, a systematic review by Robertson and Wood in 2016 showed higher RTS rates in displaced midshaft clavicle fractures after ORIF compared to a conservative treatment [15]. Therefore, the aim of this study was to address functional outcome and time until return to sport after arthroscopic assisted ORIF of isolated lateral clavicle fractures, comparing two coracoclavicular button fixation techniques (knotted vs. knotless Dog Bone ™ Button) with respect to irritation and limitation in functional outcome. It was hypothesized that LCP plating using a knotless button fixation leads to lower irritation rates after ORIF of unstable lateral clavicle fractures.

## 2. Materials and Methods

The study was approved by the institutional ethics committee and conducted according to the Declaration of Helsinki (IRB number: 350/15 s). A retrospective chart review from January 2014 to December 2018 of patients with isolated unstable or displaced lateral clavicle fractures who were operatively treated in a university level 1 trauma center was performed. Lateral clavicle fractures were assessed according to Neers’ classification. Unstable fractures or a displacement of the fracture fragments greater than 100% of the clavicle shaft were considered as indication for surgical treatment. Inclusion criteria were isolated displaced (Neer I, III) or unstable lateral clavicle fractures (Neer IIa, IIb), aged between 18 and 90 years. Delinquent patients, pregnancy, polytraumatized patients and patients under judicial care were excluded from the study. Magnetic resonance imaging was performed in case of suspected concomitant injury of the shoulder. In the case of additional trauma (concomitant fractures, rotator cuff tears etc.), patients were excluded from this study to provide a homogenous study population.

### 2.1. Surgical Technique

**Figure 1:**
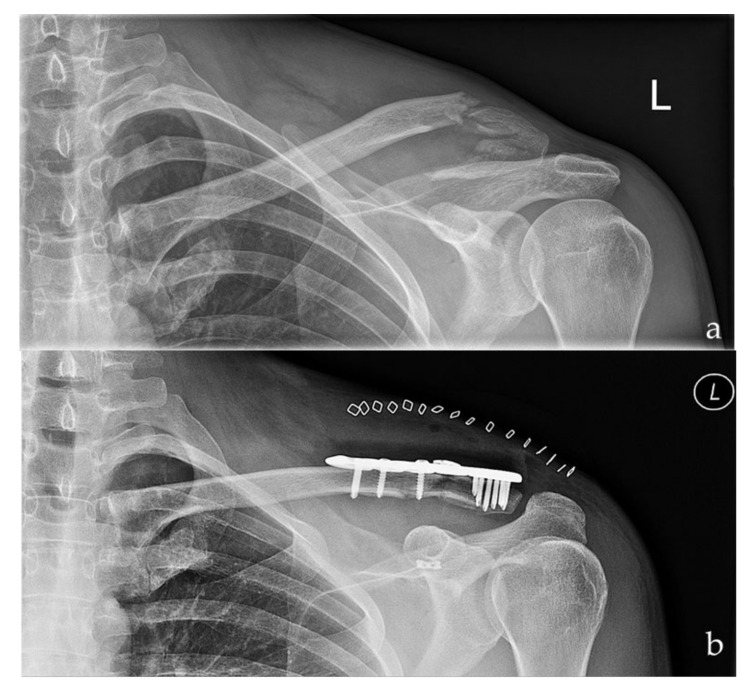
Preoperative (**a**) and postoperative (**b**) radiographs of a left sided multi-fragmentary, displaced lateral clavicle fracture (Neer IIb) after arthroscopic assisted fixation using a LCP and the knotted DogBone Technique (Group I).

**Figure 2:**
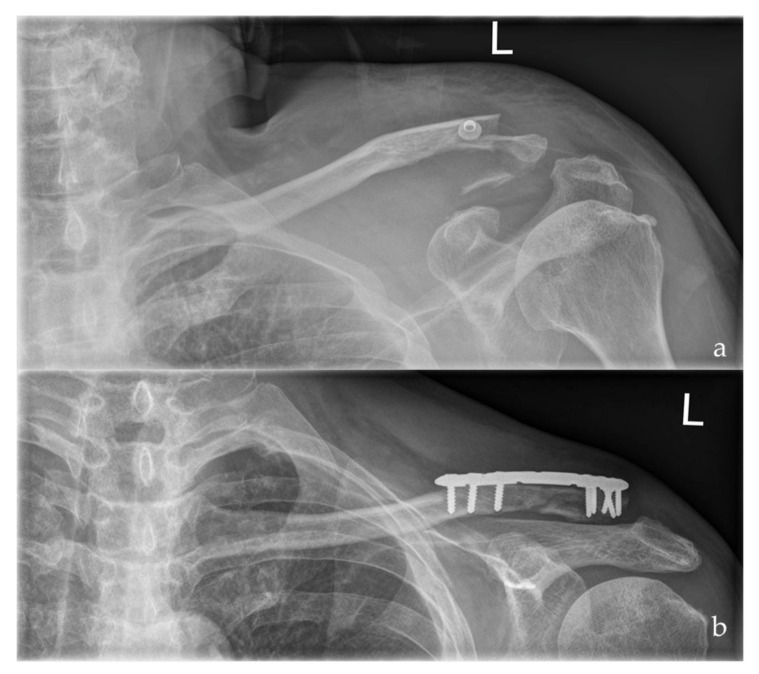
Preoperative (**a**) and postoperative (**b**) radiographs of a left sided multi-fragmentary, displaced lateral clavicle fracture (Neer IIb) after arthroscopic assisted ORIF using a LCP and the knotless DogBone Technique (Group II).

The lateral clavicle fractures were addressed using a locking compression plate (Arthrex^®^, Naples, Florida, USA) and the coracoclavicular ligament injury was taken care of either by a knotted or knotless DogBone ™ (Arthrex, Naples, Florida, USA) Button technique, respectively (Figure 1 and Figure 2). A small longitudinal skin incision was carried out to address the lateral clavicle fracture. The fracture was reduced and stabilized using a locking compression plate. A routine diagnostic arthroscopic procedure followed by preparation of the coracoid base was performed. For preparation of the base of the coracoid process care has to be taken of the subscapularis tendon which lies below and directs medially to the coracoid process. Furthermore, the glenoid labrum should not be damaged during preparation. The musculocutaneous nerve must be taken care of as it lies medial to the coracoid base, why subtle preparation has to be performed in this region. The DogBone™ Button was inserted and either knotted (Group 1) or locked using a knotless (Group 2) technique. These two techniques have been compared to each other with respect to functional outcome and irritation rates.

### 2.2. Follow up Evaluation

Routine functional and radiographic outcome was assessed 6, 12, 26 weeks postoperatively. Flexion and abduction were restricted to 30° for 2 weeks, 45° from week 3 to 4 and 60° for week 5 to 6, postoperatively. External rotation was limited for 2 weeks postoperatively. The final follow-up was performed 1 year after surgery using the American Shoulder and Elbow Score, Constant-Murley Score, Disability of Shoulder, Arm and Hand, Munich Shoulder Questionnaire, Shoulder Pain and Disability Index, respectively.

In case patients wish for implant removal, additional follow-up exams were performed until implant removal. Radiographs in two planes of the lateral clavicle were assessed to ensure completed fracture consolidation.

Return to sport was investigated in all patients. A cohort of twenty consecutive patients (*n* = 17 in group 1, *n* = 3 in group 2) participated in a specific return to sport questionnaire. Furthermore, this cohort was comprehensively reviewed for their postoperative function, duration, intensity and quantity of sports after open reduction internal fixation.

Time duration of performed sports was categorized into <30 min, >30 min, >60 min or >120 min, respectively. Intensity was categorized from 0–5 (0 = easy sportive activity, 5 professional athlete). The quantity of sportive activity assessed how often patients performed sport per week (1x/week, 2–4/week, >4 times a week). Patients were further asked for the 3 main sportive activities pre- and postoperatively.

The return to sport questionnaire includes 15 common sport activities (such as soccer, skiing, tennis, running, riding bicycle, golf etc.) including a multiple-choice answer for unmentioned sports. The three main sportive activities were assessed pre- and postoperatively and compared to each other.

### 2.3. Statistical Analysis

Statistical evaluation has been performed using Microsoft Excel 16 for Mac (Microsoft^®^) for descriptive statistics. A Mann–Whitney-*U* test was performed using SPSS 25 (IBM SPSS Statistics for Windows, NY: IBM Corp.) to identify statistically significant results within the two investigated groups. A *p*-value of <0.05 was considered statistically significant.

## 3. Results

**Figure 3:**
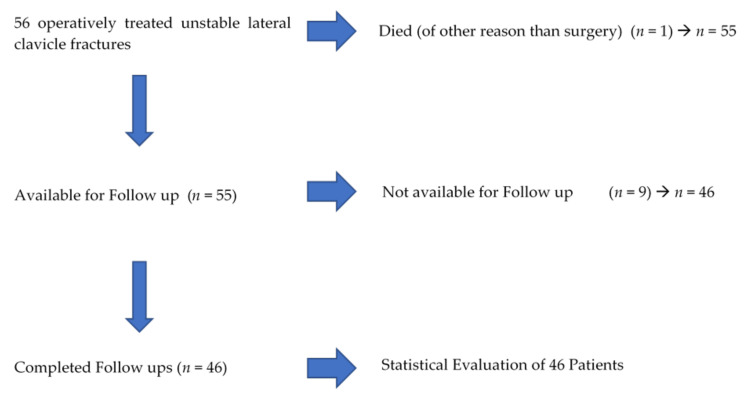
Flow chart of included/excluded patients.

Between 2014 and 2018, 56 isolated lateral clavicle fractures were operated in our institution. 46 (82.1%, 34 male, 12 female) patients with a mean age of 45.0 ± 13.9 years (18.7–80.8) participated in all follow-up examinations (Figure 3). The mean follow up was 39.5 ± 17.8 (14–71.6) months. 29 patients (63%, Group 1) were treated using the knotted DogBone™ technique, 17 with the knotless technique (37%, Group 2). A total of 18 (39.1%) fractures occurred on the right-hand side, whereas 28 (60.9%) occurred on the left. Fractures were classified as 2 type I, 17 type IIa, 26 type IIb and 1 type III lateral clavicle fractures according to Neer [3,16]. All fractures healed in the follow-up period without re-fractures, mal- or non-union.

The overall mean functional outcome one year postoperatively revealed good to excellent results. Table 1 presents the comparison of group 1 and 2 with respect to functional outcomes, without revealing statistically significant differences for the two treatment groups. The overall ASES (94.7 ± 9.8), MSQ (90.0 ± 6.0) and SPADI (96.1 ± 5.7) showed excellent results in both groups. Furthermore, the overall mean Constant-Murley Score reached 85.1 ± 8.1 representing good results. The DASH-Score showed an overall mean of 5.5 ± 8.4 representing a good result (see Table 1).

### 3.1. Limitation of Range of Motion/Irritation and Implant Removal

Overall, 39/46 (84.8%) patients were satisfied with the surgical result without having limitation on range of motion at final follow-up. However, 20/46 patients (43.5%) experienced irritation over the operated region with 19/29 (65.5%) of patients being in group 1 and only one patient (1/17, 5.9%) in group 2 (*p* = 0.004, s. (statistically significant)). Overall, 20 implant removals were performed after a mean of 18.8 ± 10.6 months. A total of 14/29 (48.3%) implant removals were performed in group 1, whereas 6/17 (35.3%) in group 2 (*p* = 0.122, n.s. (not statistically significant)).

### 3.2. Return to Sports

A total of 33/46 (71.7%) patients performed sports prior to their injury. All of them (100%) returned to sports after ORIF. The three main sport activities preoperatively were cycling (*n* = 17, 51.1%), jogging/running (*n* = 13, 39.4%) and skiing (*n* = 13, 39.4%). Postoperatively the three mainly performed sports were cycling (*n* = 17, 51.1%), skiing (*n* = 14, 42.4%) and jogging/running (*n* = 13, 39.4%).

The first 20 (15 male, 5 female) consecutive patients with a mean age of 46.4 ± 11.4 years (21–67 years) participated in a specific return to sports questionnaire.

These 20 patients presented with eight right and 12 left clavicle fractures, whereas 17 right hand and three left hand dominant patients were observed. A total of 17 patients were operated using the knotted DogBone™ technique and only three with the knotless technique, respectively. Mean time interval until return to sport was 4.6 (3–9) months. After 3 months, 11 patients returned to sport, eight patients after 6 and one patient after 9 months. The time period until return to sport did not reveal statistically significant differences for gender or age (n.s.). The used technique (knotted vs. knotless) did not show statistically significant results with respect to return to sport (n.s.).

A total of 11/17 (64.7%) of patients who were operated using the knotted technique reported irritation during daily activities or sports and 4/20 (20%) of patients reported postoperative restriction with respect to range of motion 1 year postoperatively.

Quantity of sports was 1x/week in 10 patients, 2–4x times/week in 9 patients and >4 times/week in one patient. A total of 17 patients performed the same amount of sport postoperatively, three patients increased their activity from 1 to 2–4x times/week.

The preoperative intensity of performed sport was measured from 0–5 (0 = easy sportive activity, 5 professional athlete). Six patients reported level 0, 3 patients level 1, 3 patients level 2, 5 patients level 3 and 3 patients level 4. None of the patients performed professional sports. Three patients increased their intensity postoperatively from level 1 to 2, two patients decreased their activity from 2 to 1 and one patient from 1 to 0, respectively. Time duration of performed sports was measured as follows: 30 min, >30 min, >60 min, >120 min. Preoperatively, one patient trained for <30 min, six patients >30 min, 9 patients >60 min and four patients >120 min. A total of 15 patients performed the same amount of time, two patients decreased the duration from >60 to >30 min and three patients increased it from >30 to >60 min.

With respect to range of motion (flexion, abduction, internal/external rotation) only internal rotation showed a minor restriction in 9 patients 1 year postoperatively (MSQ 8/10 points for internal rotation). Flexion, abduction as well as external rotation showed no limitations 1 year postoperatively.

## 4. Discussion

The main findings of the present study were that ORIF with additional button fixation of unstable lateral clavicle fracture show good to excellent clinical results and a high return to sports rate. Knotless button fixation significantly decreased postoperative soft-tissue irritation around the clavicle and, even though not significant, the need for implant removal. The comparison of the used techniques in this study, to the best of our knowledge, is the first in literature. The RTS in young and active patients (not professional athletes) has also not been well depicted in literature until recently.

Unstable or severely displaced lateral clavicle fractures need to be treated operatively. Numerous surgical techniques have been described in the literature [17,18,19,20]. In this context, hook plating was routinely used for this entity, leading to controverse results with respect to irritation of the skin in contrast to good to excellent functional outcome [9,20]. The arthroscopic assisted approach was established in the early 2000s’ and has been developed ever since, showing promising results with regard to functional outcome and low implant failure rates [6,18,19,21,22].

In 2014, Beirer et al. depicted that in Neer type II fractures of the lateral clavicle additional surgical stabilization of the coracoclavicular ligaments is necessary since this type of fracture results in acromioclavicular instability [1]. Additionally, biomechanical studies revealed high fracture stability using locking compression plates combined with coracoclavicular stabilization [12]. Another advantage of the arthroscopic approach compared to hook plating is the fact that concomitant glenohumeral injuries, detected in up to 46% of lateral clavicle fractures, can be additionally addressed [23]. Furthermore, the irritation rates in patients treated using hook plates showed inferior functional outcomes compared to patients treated arthroscopically [2]. However, irritation due to inserted implants still represents a problem in our experience, even in patients treated by the arthroscopic assisted technique. Therefore, the presented study focused on a comparison of two versions of an arthroscopic assisted technique, knotted vs. knotless. Patients treated using the knotted technique often complained about restriction or pain after operative treatment. Since there is a lack of information in the literature, we reached out to gain information comparing the knotted and knotless technique. In this context, Stegeman et al. demonstrated an 11-fold higher risk of major complications using hook plating, compared to other surgical techniques in treating unstable lateral clavicle fractures [2]. The mandatory need of implant removal of the hook plate is one reason why we changed our protocol to an arthroscopic assisted technique. Secondly, additional damage to the acromioclavicular joint may occur when inserting, as well as removing, the hook plate. However, the hook plate was also developed over the years to reduce additional damage to the acromioclavicular joint [9,24]. Menge et al. showed lower irritation rates using an arthroscopic assisted approach when compared to earlier open techniques in acromioclavicular instability [25]. However, to distinguish irritation caused by locking compression plates, as well as by inserted fibers, has been troublesome. From a clinical point of view there exists no test allowing for a differentiation between plate irritation and irritation due to inserted fibers. However, in earlier revision surgery, incomplete closure of the trapezodeltoid fascia was considered as one possible reason for subcutaneous irritation of fibers. Therefore, the presented study reached out to address this issue accompanied by a return to sport questionnaire.

### 4.1. Irritation Rates

The presented high irritation rates (19/29, 65.5%), as well as the higher number of implant removal in group 1 (knotted technique, 14/29, 48.3%) indicate irritation rather from the Fiberwire than from the locking compression plate. We noticed a relatively high implant removal rate in our hospital compared to the results presented by Fleming et al. (*n* = 2/19, 10.5%) and Sautet et al. (*n* = 2/14, 14.3%) [13,17]. In a recent study, we found high implant removal rates in patients after displaced clavicle fractures due to manifold reasons [26]. High irritation rates, as well as psychological reasons (pondering about implanted material), were the main reasons for a patients’ wish for implant removal after ORIF of displaced clavicle fractures in our cohort [26].

### 4.2. Return to Sports

The time duration until return to sport is crucial for professional athletes. Yet this presents an important factor for active patients as well. Robertson et al. presented a higher return to sport rate after operative treatment of lateral clavicle fractures compared to a non-operative treatment [15]. In our cohort, we reached out for a return to sport questionnaire to gain information with regard to irritation, functional outcome, as well as the time until return to sport. Of all 33 patients who performed sports prior to their injury, 100% returned to sport after surgery. Twenty consecutive patients were investigated utilizing a return to sport questionnaire, which is routinely used in our department for professional athletes. The first twenty patients who gave written informed consent to complete the questionnaire were enclosed for this investigation. Patients were asked for the time until return to sport, the level of activity (1x week up to more than 4x/week), time duration of sport (<30 up to >120 min/session) as well as intensity of sport activities (0 = easy sportive activity–5 professional athlete). Furthermore, the main three sportive activities were identified and compared pre- and postoperatively. Neither age (*p* = 0.736), nor gender (*p* = 0.612) or used surgery technique (*p* = 0.489) showed statistically significant differences in regard to return to sport, respectively. We assume the level of activity should not always be connected to patients age rather than to the patients’ functional demands. The time until return to sport (mean of 4.6 months, range: 3–9 months) was comparable to other surgical treatment regimen of the shoulder girdle. In a systematic review, Abdul-Rassoul et al. found 91.7% of patients who did return to sports after a mean of 5.9 months after arthroscopic bankart repair [27]. Verstift et al. reported on a mean time until return to sport of four months after surgical intervention for acromioclavicular instability in their systematic review of 462 patients [28].

### 4.3. Arthroscopic Assisted Operative Treatment

The arthroscopic assisted approach represents a safe and viable technique with a relatively low complication rate compared to earlier (open) techniques [2,6,13]. Kapicioglu reported on coracoid process fractures which represents an important complication using the arthroscopic assisted technique [6]. In the case of multiple drillings through the coracoid process, the likelihood of fracture increases [25]. We did not encounter fractures or re-fractures in our cohort. Tunnel positioning should always strive to be in the center-position (antero-posterior) of the clavicle to minimize the risk of re-fractures. Multiple drill holes are, furthermore, in favor for construct failure, giving this surgical technique a certain learning curve.

### 4.4. Functional Outcome

One issue to compare ROM, as well as various scores in the current literature, are the varying scores and the rather low amount of studies on the presented technique [6,13,25]. The presented Scores (ASES, Constant Score, DASH, MSQ and SPADI) in this study are routinely used in our department. The mean functional outcome after arthroscopic assisted lateral clavicle repair was good considering the Constant-Murley Score (85.1 ± 8.1), as well as excellent referring to the ASES (94.7 ± 9.8), MSQ (90.9 ± 7.2) and SPADI (96.1 ± 5.7) in our cohort when compared to recent results by Kapicioglu et al. [6]. They presented an ASES Score of 92.6 ± 3.2 and a Constant-Murley Score of 96.2 ± 2.4 when addressing lateral clavicle fractures with an all arthroscopic coracoclavicular button fixation technique. Sautet et al. could also show excellent results (Constant Score 91%, SSV 95%) when using an arthroscopic assisted technique [13]. Minor restriction while performing internal rotation was reported by 9 patients from our cohort 1 year postoperatively. However, the presented results did not reveal a statistically significant value (*p* = *0*.201). We assume the preparation of the (sub)-coracoid region resulting in postoperative adhesions responsible for this issue.

## 5. Limitations

The first limitation is the relatively small patient number of 46 patients. However, we only included patients with isolated lateral clavicle fractures. Secondly, the return to sports questionnaire was assessed by only 20 patients, yet, to the best of our knowledge, current literature does not provide more information with respect to this field. Thirdly, the disparate distribution of used techniques (knotted *n* = 3 and knotless *n* = 17) explains why the statistical evaluation for this hypothesis is of limited value. Another limitation is the change of sportive activities one year postoperatively, which are, in most cases, not treatment related. However, the change of sportive activities in the majority of reported patients are not attributable to surgical treatment.

## 6. Conclusions

Locking compression plating and coracoclavicular fixation using a knotless Dogbone™ for the treatment of lateral clavicle fractures technique is a safe and viable method, which provides good to excellent functional outcomes and a fast return to sport (mean of 4.6 months). Irritation rates, as well as implant removal rates, were lower in the knotless group compared to the knotted technique, yet without statistical significance.

## Figures and Tables

**Table 1:** Comparison of the functional outcome one year postoperative after arthroscopic assisted ORIF of unstable/displaced lateral clavicle fractures.

	Group 1	Group 2	*p*-Value
**ASES**	94.71 ± 8.1	94.69 ± 5.6	0.160 (n.s.)
**Constant Score**	84.89 ± 8.8	85.52 ± 6.9	0.422 (n.s.)
**DASH**	5.44 ± 9.5	5.58 ± 6.2	0.360 (n.s.)
**MSQ**	91.10 ± 8.1	90.58 ± 5.6	0.466 (n.s.)
**SPADI**	90.06 ± 6.5	90.05 ± 4.0	0.178 (n.s.)
**Abduction**	168.5 ± 23.8	157.5 ± 27.0	0.146 (n.s)
**Flexion**	165.4 ± 27.3	153.8 ± 27.8	0.048 (s.)
**Internal Rotation**	90 ± 15.4	85.9 ± 16.3	0.201 (n.s)
**External Rotation**	40.9 ± 14.5	40.6 ± 17.2	0.177 (n.s)

ASES: American Shoulder and Elbow Score; DASH: Disabilities of Arm, Shoulder and Hand; MSQ: Munich Shoulder Questionnaire; SPADI: Shoulder Pain and Disability Index; n.s.: not statistically significant; s.: statistically significant.

## Data Availability

Source entry data can be obtained on reasonable request via the first author.
